# LocoMMotion: a prospective, non-interventional, multinational study of real-life current standards of care in patients with relapsed and/or refractory multiple myeloma

**DOI:** 10.1038/s41375-022-01531-2

**Published:** 2022-03-24

**Authors:** Maria-Victoria Mateos, Katja Weisel, Valerio De Stefano, Hartmut Goldschmidt, Michel Delforge, Mohamad Mohty, Michele Cavo, Ravi Vij, Joanne Lindsey-Hill, Dominik Dytfeld, Emanuele Angelucci, Aurore Perrot, Reuben Benjamin, Niels W. C. J. van de Donk, Enrique M. Ocio, Christof Scheid, Francesca Gay, Wilfried Roeloffzen, Paula Rodriguez-Otero, Annemiek Broijl, Anna Potamianou, Caline Sakabedoyan, Maria Semerjian, Sofia Keim, Vadim Strulev, Jordan M. Schecter, Martin Vogel, Robert Wapenaar, Tonia Nesheiwat, Jesus San-Miguel, Pieter Sonneveld, Hermann Einsele, Philippe Moreau

**Affiliations:** 1 University Hospital of Salamanca/IBSAL/CIC, Salamanca, Spain; 2 University Medical Center Hamburg-Eppendorf, Hamburg, Germany; 3 Section of Hematology, Catholic University, Fondazione Policlinico A. Gemelli, IRCCS, Rome, Italy; 4 University Hospital Heidelberg, Heidelberg, Germany; 5 University Hospitals (UZ) Leuven, Leuven, Belgium; 6 Service d’Hematologie Clinique et Therapie Cellulaire, Sorbonne University, INSERM UMRs 938, Paris, France; 7 IRCCS Azienda Ospedaliero-Universitaria di Bologna, Istituto di Ematologia Seràgnoli, Bologna University School of Medicine, Bologna, Italy; 8 Washington University School of Medicine, St. Louis, MO USA; 9 Nottinghamshire University Hospitals NHS Trust, Nottingham, UK; 10 Poznań University of Medical Sciences, Poznań, Poland; 11 Hematology and Transplant Center, IRCCS Ospedale Policlinico San Martino, Genova, Italy; 12 Centre Hospitalier Universitaire de Toulouse, Service d’Hématologie, Toulouse, France; 13 Department of Haematology, King’s College Hospital, London and School of Cancer and Pharmaceutical Sciences, King’s College London, London, UK; 14 Amsterdam UMC, Vrije Universiteit Amsterdam, Amsterdam, Netherlands; 15 Hospital Universitario Marqués de Valdecilla (IDIVAL), Universidad de Cantabria, Santander, Spain; 16 University of Cologne, Cologne, Germany; 17 University of Torino, Torino, Italy; 18 University Medical Center Groningen, Groningen, Netherlands; 19 Clínica Universidad de Navarra, CIMA, CIBERONC, IDISNA, Pamplona, Spain; 20 Erasmus MC Cancer Institute, Rotterdam, Netherlands; 21 Janssen-Cilag, Neuss, Germany; 22 Janssen-Cilag, Beirut, Lebanon; 23 Janssen-Cilag, Issy-les-Moulineaux, France; 24 Janssen-Cilag, Porto Salvo, Portugal; 25 Janssen Pharmaceutica NV, Beerse, Belgium; 26 Janssen R&D, Raritan, NJ USA; 27 Janssen Global Services, LLC, Raritan, NJ USA; 28 Janssen-Cilag BV, Breda, Netherlands; 29 Legend Biotech USA Inc, Piscataway, NJ USA; 30 Universitätsklinikum Würzburg, Medizinische Klinik und Poliklinik II, Würzburg, Germany; 31 University Hospital Hotel-Dieu, Nantes, France

**Keywords:** Myeloma, Targeted therapies

## Abstract

Despite treatment advances, patients with multiple myeloma (MM) often progress through standard drug classes including proteasome inhibitors (PIs), immunomodulatory drugs (IMiDs), and anti-CD38 monoclonal antibodies (mAbs). LocoMMotion (ClinicalTrials.gov identifier: NCT04035226) is the first prospective study of real-life standard of care (SOC) in triple-class exposed (received at least a PI, IMiD, and anti-CD38 mAb) patients with relapsed/refractory MM (RRMM). Patients (*N* = 248; ECOG performance status of 0–1, ≥3 prior lines of therapy or double refractory to a PI and IMiD) were treated with median 4.0 (range, 1–20) cycles of SOC therapy. Overall response rate was 29.8% (95% CI: 24.2–36.0). Median progression-free survival (PFS) and median overall survival (OS) were 4.6 (95% CI: 3.9–5.6) and 12.4 months (95% CI: 10.3–NE). Treatment-emergent adverse events (TEAEs) were reported in 83.5% of patients (52.8% grade 3/4). Altogether, 107 deaths occurred, due to progressive disease (*n* = 74), TEAEs (*n* = 19), and other reasons (*n* = 14). The 92 varied regimens utilized demonstrate a lack of clear SOC for heavily pretreated, triple-class exposed patients with RRMM in real-world practice and result in poor outcomes. This supports a need for new treatments with novel mechanisms of action.

## Introduction

Despite advances in medical treatment that have improved survival, multiple myeloma (MM) remains incurable [1]. Most patients with MM eventually progress or become refractory to treatment with standard drug classes including proteasome inhibitors (PIs), immunomodulatory drugs (IMiDs), anti-CD38 monoclonal antibodies (mAbs), and others [2]. Currently, there is an incomplete understanding of how heavily pretreated triple-class exposed (received at least a PI, IMiD, and anti-CD38 mAb) MM patients are treated in a real-world setting and their outcomes.

Findings from the MAMMOTH study, a retrospective study of treatment outcomes in patients in the United States with MM, reported an overall response rate (ORR) of 31% with median overall survival (OS) of 9.3 months in refractory patients who were triple-class exposed [3]. These data highlight the poor outcomes in this heavily pretreated group of patients and suggest the need for more effective therapies.

However, to date there have been no multinational prospective studies examining outcomes of the standard of care (SOC) used in everyday clinical practice for heavily pretreated triple-class exposed patients. Here, we present results from the LocoMMotion study (NCT04035226), the first prospective, non-interventional, multinational study to assess the effectiveness of real-life SOC treatments in patients with RRMM who have been previously treated with a PI, an IMiD, and an anti-CD38 mAb.

## Subjects and Methods

### Study design and treatment

LocoMMotion is an ongoing, prospective, non-interventional study detailing the use of real-life current SOC in the treatment of RRMM patients who have received ≥3 prior lines of therapy (LOT) or were double refractory to a PI and an IMiD; received a PI, IMiD, and anti-CD38 mAb; and have documented disease progression during or after their last LOT. There were no exclusion criteria for prior therapies received by patients. It was conducted across 76 sites including 63 in Europe (Belgium, France, Germany, Italy, Netherlands, Poland, Russia, Spain, and the United Kingdom) and 13 in the United States; 248 patients were enrolled between August 2, 2019 and October 26, 2020. Eligible patients were ≥18 years old and had a documented diagnosis of MM per International Myeloma Working Group (IMWG) criteria [4–6]; measurable disease, assessed by M-protein (≥1.0 g/dL [serum] or ≥ 200 mg/24 h [urine]) or serum free light chain (≥10 mg/dL and abnormal ratio), and an Eastern Cooperative Oncology Group performance status (ECOG PS) of 0 or 1.

The study included a 28-day screening phase (including first day of SOC treatment, where baseline patient and disease characteristics were collected), an SOC treatment phase (time from the first day of SOC treatment until progressive disease, unacceptable toxicity, or initiation of subsequent antimyeloma therapy, where efficacy and safety data were collected), and a follow-up phase until study completion (where patients were followed for survival and subsequent therapies). Study completion was defined as 24 months after first dose of the last patient enrolled in the study. Patient-reported outcomes were also collected (not reported in this article). SOC treatments were defined as those used in local clinical practice for the treatment of adult patients with RRMM, experimental drugs were not allowed. A Response Review Committee (RRC) composed of three leading hematologists in the field of MM reassessed responses per IMWG criteria, in a blinded manner, to ensure consistency of the assessments.

This study was conducted in accordance with the declaration of Helsinki. All patients provided written informed consent. An independent ethics committee/institutional review board at each center approved the study protocol.

To account for potential missing assessments in real-world clinical practice that are required for response evaluation, per strict IMWG criteria [6], additional measures were applied, as follows. The study was designed to collect all available data necessary for response evaluation at a minimum of each cycle of treatment (including serum protein electrophoresis, serum immunofixation electrophoresis, serum-free light chains, serum quantitative immunoglobulins, 24-hour urine M-protein quantitation by electrophoresis, urine immunofixation electrophoresis, as well as plasmacytomas, bone lesion, and bone marrow assessments). RRC reassessment of the response for each cycle of treatment was blinded to ensure consistency of assessment. The RRC used the strict IMWG criteria [6] with limited flexibility to mitigate potential missing information and avoid underestimation of the response.

### Endpoints and assessments

The primary endpoint was ORR, defined as the proportion of patients who achieved partial response (PR) or better according to the IMWG criteria, as assessed by the RRC. Secondary clinical assessments included rates of stringent complete response (sCR), complete response (CR), very good partial response (VGPR), duration of response (DOR), progression-free survival (PFS), and OS. Safety assessments included incidence and severity of treatment-emergent adverse events (TEAEs). Incidence of secondary primary malignancies was also collected.

### Statistical analyses

Given the observational nature of the study, no direct hypothesis was tested; sample size was based on clinically acceptable precision of the 95% confidence interval (CI) for the primary objective. When the sample size was 230, using the large sample normal approximation, the width of a two-sided 95% CI varied from 0.107 to 0.130 for an expected proportion varying from 0.20 to 0.40. A sample size of 230 patients was assumed sufficient to investigate secondary objectives. Continuous variables were summarized using the number of observations, mean, standard deviation, coefficient of variation, median, and range. Time-to-event data were summarized by 25th, 50th, and 75th percentiles with two-sided 95% CIs. Categorical values were summarized using the number of observations and percentages.

## Results

### Patients

**Fig. 1:**
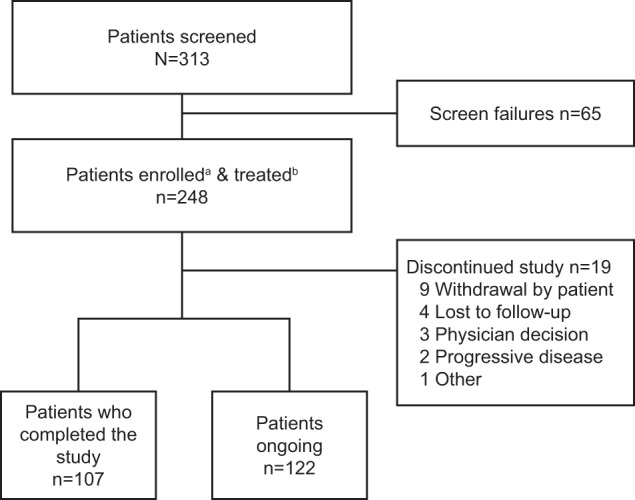
Study disposition. ^a^Enrolled patients are those who signed informed consent and were formally enrolled into the study. ^b^Treated patients are those who were enrolled in the study and received at least one standard of care treatment.

Of the 248 patients enrolled and treated, 225 (90.7%) were from Europe and 23 (9.3%) from the United States. As of the data cut-off date of May 21, 2021, representing a median follow-up of 11.01 months (range, 0.1–19.2), 107 (43.1%) patients had completed the study due to death, 122 (49.2%) patients were ongoing, and 19 (7.7%) patients had discontinued (Fig. 1).

Patient characteristics are summarized in Table 1. Median age was 68 years (range, 41–89), and 135 patients (54.4%) were male; 180 (72.9%) had a baseline ECOG PS score of 1. Median time since initial MM diagnosis was 6.3 years (range, 0.3–22.8). Patients had received a median of 4.0 prior LOTs (range, 2–13); 16 (6.5%) patients had received 2 prior LOTs and were eligible for this study due to double-refractoriness to PI and IMiD. Nearly half of the patients (122; 49.2%) had received ≥5 prior LOTs. All patients were triple-class exposed, 183 (73.8%) were triple-class refractory, and 230 (92.7%) were refractory to their last line of therapy. Seventy (32.3%) patients each presented as International Staging System (ISS) stages I and II at study entry, and 77 (35.5%) as stage III. Extramedullary plasmacytomas were present in 33 (13.3%) patients. Overall, 160 patients (64.5%) had undergone previous stem cell transplant (160 [64.5%] autologous, 11 [4.4%] allogeneic).

**Table 1:** Baseline characteristics.

Characteristic	*N* = 248
Median age (range), years	68 (41–89)
Male, *n* (%)	135 (54.4)
Geographic region, *n* (%)	
United States	23 (9.3)
Europe	225 (90.7)
Race,^a^ *n* (%)	*N* = 190
White	182 (95.8)
Black	5 (2.6)
Other	1 (0.5)
Unknown	2 (1.1)
Baseline ECOG score,^b^ *n* (%)	
0	63 (25.5)
1	180 (72.9)
2	3 (1.2)
3	1 (0.4)
Time from initial MM diagnosis, median (range) years	6.3 (0.3–22.8)
Number of prior lines of therapy, median (range)	4.0 (2–13)
Prior lines of therapy, *n* (%)	
2	16 (6.5)
3	48 (19.4)
4	62 (25.0)
≥5	122 (49.2)
ISS Stage (at study entry), *n* (%)	
I	70 (32.3)
II	70 (32.3)
III	77 (35.5)
Presence of extramedullary plasmacytomas	
Yes	33 (13.3)
No	215 (86.7)
Type of measurable disease	
Serum only	123 (49.6)
Serum and urine	19 (7.7)
Urine only	22 (8.9)
Serum free light chain	82 (33.1)
Not evaluable	2 (0.8)
Previous stem cell transplant, *n* (%)	
Autologous	160 (64.5)
Allogeneic	11 (4.4)
LDH (U/L)	
≤245	114 (61.3)
>245	72 (38.7)
Creatinine clearance (mL/min)	
≤60	94 (40.0)
>60	141 (60.0)
Triple-class exposed,^c^ *n* (%)	248 (100)
Refractory status, *n* (%)	
Any PI	197 (79.4)
Any IMiD	234 (94.4)
Any anti-CD38 mAb	228 (91.9)
Triple-class refractory	183 (73.8)
Penta-drug refractory	44 (17.7)
Refractory to last line of prior therapy, n (%)	230 (92.7)

*ECOG* Eastern Cooperative Oncology Group, *IMiD* immunomodulatory drug, *ISS* International Staging System, *LDH* lactate dehydrogenase, *mAb* monoclonal antibody, *MM* multiple myeloma, *PI* proteasome inhibitor.

^a^Race was not reported for 58 patients.

^b^Screening ECOG scores were 0 or 1 only.

^c^Any PI, any IMiD, and any anti-CD38 mAb.

### Treatment summary

SOC treatment regimens are summarized in Table 2. Overall, 92 unique SOC treatment regimens were used in the enrolled population, including corticosteroids, PIs, IMiDs, alkylating agents, and anti-CD38 mAbs and various combinations thereof, with 160 (64.5%) patients treated with a combination of ≥3 drugs (Supplementary Table 1). The most frequent used PI, IMiD, and anti-CD38 mAb were carfilzomib (25.4%), pomalidomide (29.8%), and daratumumab (9.3%), respectively. Patients received a median of 4.0 (range, 1–20) cycles of SOC therapy and spent a median of 3.9 months (range, <1.0–18.0) on treatment. Six (2.4%) patients underwent autologous transplant, and no patients underwent allogeneic transplant. The most common reason for treatment discontinuation was disease progression in 112 (45.2%) patients.

**Table 2:** Antimyeloma standard of care therapy.

SOC treatment, *n* (%)^a^	*N* = 248
Glucocorticoid	220 (88.7)
PI	133 (53.6)
Carfilzomib	63 (25.4)
Bortezomib	48 (19.4)
Ixazomib	22 (8.9)
IMiD	117 (47.2)
Pomalidomide	74 (29.8)
Lenalidomide	36 (14.5)
Thalidomide	7 (2.8)
Alkylating agent	107 (43.1)
Cyclophosphamide	79 (31.9)
Bendamustine	16 (6.5)
Melphalan	15 (6.0)
Anti-CD38 monoclonal antibody	24 (9.7)
Daratumumab	23 (9.3)
Isatuximab	1 (0.4)
Anthracyclines	18 (7.3)
Topoisomerase inhibitor	16 (6.5)
Other antineoplastic agent^b^	15 (6.0)
Histone deacetylase inhibitor	12 (4.8)
Anti-SLAMF7 monoclonal antibody	9 (3.6)
BCMA-targeted antibody-drug conjugate	7 (2.8)
Bcl-2 inhibitor	6 (2.4)
Autologous stem cell transplant	6 (2.4)
Mitotic inhibitor	2 (0.8)
Selective inhibitor of nuclear export	2 (0.8)

*BCMA* B-cell maturation antigen, *Bcl* B-cell lymphoma, *IMiD* immunomodulatory drug, *PI* proteasome inhibitor, *SLAM* signaling lymphocytic activation molecule, *SOC* standard of care.

^a^There was a large amount of heterogeneity in the combination therapies. Patients may have been counted in more than one regimen.

^b^Other antineoplastic agents included cisplatin and rituximab.

At the time of the data cut-off, 123 (49.6%) patients were exposed to subsequent antimyeloma therapies (Supplementary Table 2). Seventy-eight (31.5%) patients received 1 subsequent LOT and 45 (18.2%) patients received >1 subsequent LOT. Between 2020 and 2021, 99 unique regimens were used in subsequent LOTs, reflecting the existing variety of real-life antimyeloma treatments and absence of preferred SOC treatment in this population.

### Efficacy

**Fig. 2:**
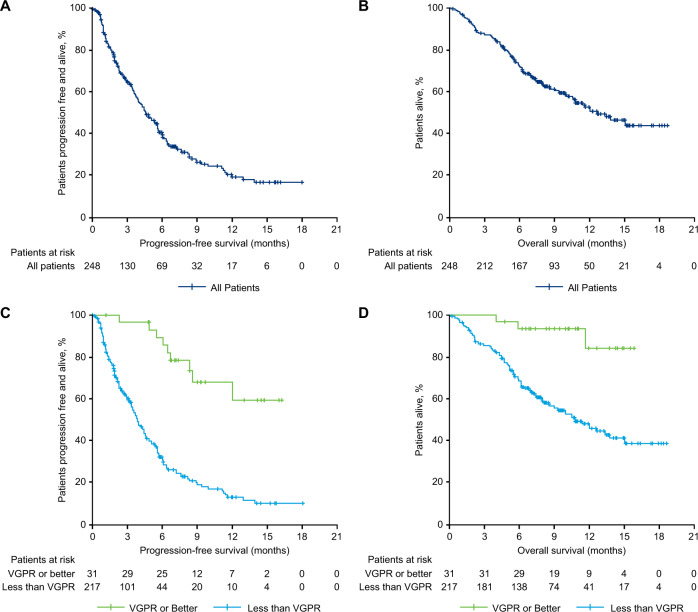
Kaplan–Meier plots for survival outcomes. Progression-free survival and overall survival based on RRC assessment in all patients (**A**, **B**) and in patients who achieved VGPR or better versus those who did not (**C**, **D**). *RRC* Response Review Committee, *SOC* standard of care, *VGPR* very good partial response.

The ORR for patients treated with real-life SOC therapy was 29.8% (95% CI: 24.2–36.0) (Table 3). Median DOR was 7.4 months (95% CI: 4.7–12.5). None of the patients achieved sCR; 1 patient (0.4%) achieved CR, 30 patients (12.1%) achieved VGPR, 43 (17.3%) achieved PR, 13 patients (5.2%) achieved minimal response, 77 patients (31.0%) had stable disease, and 46 patients (18.5%) had progressive disease. Thirty-eight (15.3%) patients were considered not evaluable, of which 14 were due to death (<2 months after starting SOC therapy) and 12 to stopping or switching SOC therapy (most often due to rapid disease progression, based on investigator analysis). Median PFS was 4.6 months (95% CI: 3.9–5.6), and median OS was 12.4 months (95% CI: 10.28–NE; Fig. 2A, B). The 12-month PFS and OS rates were 19.9% (95% CI: 13.6–27.0) and 51.8% (95% CI: 44.1–58.8), respectively.

**Table 3:** Response to standard of care treatment.

Variable	Total (*N* = 248)
Overall response rate, % (95% CI)	29.8 (24.2–36.0)
Best response, rate, %	
Stringent complete response	0
Complete response	0.4
Very good partial response	12.1
Partial response	17.3
Minimal response	5.2
Stable disease	31.0
Progressive disease	18.5
Not evaluable	15.3
Median duration of response (95% CI), months	7.4 (4.7–12.5)
Median time to first response (range), months	1.9 (0.7–9.5)
Median time to best response (range), months	2.4 (0.7–12.2)

*CI* confidence interval.

Patients who did not achieve VGPR had a median DOR of 4.5 months (95% CI: 3.5–7.3), a median PFS of 3.9 months (95% CI: 3.4–4.6), and a median OS of 10.9 months (95% CI: 8.4–14.2). For the 31 patients who achieved VGPR or better, median DOR (95% CI: 7.7–NE) and median OS (95% CI: NE–NE) were not estimable, and median PFS was not reached (95% CI: 8.54–NE; Fig. 2C, D). Patients who were triple-class refractory at baseline (*n* = 183) had an ORR of 25.1% (95% CI: 19.0–32.1), median DOR of 4.5 months (95% CI: 3.7–NE), median PFS of 3.9 months (95% CI: 3.4–4.6), and median OS of 11.1 months (95% CI: 8.8–14.2). Patients who were not triple-class refractory (*n* = 65) had an ORR of 43.1% (95% CI: 30.8–56.0), median DOR of 9.1 months (95% CI: 7.3–NE), median PFS of 8.2 months (95% CI: 5.7–12.0), and median OS was not estimable (95% CI: 12.4–NE).

### Safety

Within routine clinical practice, TEAEs were reported in 207 (83.5%) patients, with grade 3/4 TEAEs in 131 (52.8%) patients (Table 4). The most common hematologic AEs (any grade) were anemia (25.8%), thrombocytopenia (23.0%), and neutropenia (15.7%), and the most common grade 3/4 hematologic TEAEs were thrombocytopenia (17.7%), neutropenia (13.3%), and anemia (10.9%; Table 5). Overall, grade 3/4 cytopenia was reported in 85 (34.3%) patients; however, when the incidence of this TEAE was derived from laboratory data, grade 3/4 cytopenia was observed in 158 (64.8%) patients (Supplementary Table 3). This twofold discrepancy between reported cytopenic adverse events and toxicities derived from the laboratory data suggest an overall underreporting of adverse events in this study. The most common (≥10%) non-hematologic AEs of any grade were infections/infestations (28.6%), nervous system disorders (19.8%), diarrhea (15.3%), metabolism/nutrition disorders (12.5%), pyrexia (12.5%), fatigue (12.1%), and dyspnea (11.3%; Table 5). No non-hematologic TEAEs were observed at grade 3/4 at a rate of ≥10%. Second primary malignancies were reported in 6 patients. A total of 107 (43.1%) patients had died by the time of data cut-off, with disease progression being the leading cause of death (*n* = 74; 29.8%). Nineteen (7.7%) patients died due to TEAEs during the study, most commonly due to infection (*n* = 11).

**Table 4:** Severity of standard of care treatment-emergent adverse events.

TEAE, *n* (%)^a^	*N* = 248
Any TEAE	207 (83.5)
Any serious TEAE	84 (33.9)
Maximum severity of TEAE	
Grade 1	16 (6.5)
Grade 2	52 (21.0)
Grade 3	78 (31.5)
Grade 4	44 (17.7)
Grade 5	17 (6.9)
TEAE with outcome death	19 (7.7)

*TEAE* treatment-emergent adverse event.

^a^Percentages are calculated with the all-treated analysis set as denominator.

**Table 5:** Hematologic and non-hematologic treatment-emergent adverse events.

TEAE	Total (*N* = 248)	
	Any grade, *n* (%)^a^	Grade 3/4, *n* (%)^a^
Hematologic TEAEs^b^		
Total patients with hematologic TEAE	106 (42.7)	85 (34.3)
Anemia	64 (25.8)	27 (10.9)
Thrombocytopenia	57 (23.0)	44 (17.7)
Neutropenia	39 (15.7)	33 (13.3)
Leukopenia	18 (7.3)	12 (4.8)
Lymphopenia	16 (6.5)	14 (5.6)
Non-hematologic TEAEs^b^		
Infections and infestations	71 (28.6)	16 (6.5)
Nervous system disorders	49 (19.8)	8 (3.2)
General disorders and administration site conditions		
Pyrexia	31 (12.5)	6 (2.4)
Fatigue	30 (12.1)	2 (0.8)
Asthenia	23 (9.3)	2 (1.2)
Peripheral edema	19 (7.7)	1 (0.4)
Gastrointestinal disorders		
Diarrhea	38 (15.3)	2 (0.8)
Nausea	23 (9.3)	3 (1.2)
Constipation	14 (5.6)	0
Vomiting	14 (5.6)	2 (0.8)
Metabolism and nutrition disorders	31 (12.5)	9 (3.6)
Musculoskeletal and connective tissue disorders		
Back pain	20 (8.1)	4 (1.6)
Arthralgia	15 (6.0)	3 (1.2)
Respiratory, thoracic, and mediastinal disorders		
Dyspnea	28 (11.3)	6 (2.4)
Investigations	25 (10.1)	6 (2.4)
Psychiatric disorders	22 (8.9)	3 (1.2)
Renal and urinary disorders	22 (8.9)	13 (5.2)
Injury, poisoning and procedural complications	21 (8.5)	6 (2.4)
Skin and subcutaneous tissue disorders	20 (8.1)	1 (0.4)
Cardiac disorders	18 (7.3)	9 (3.6)
Vascular disorders	18 (7.3)	7 (2.8)

*TEAE* treatment-emergent adverse event.

^a^Percentages are calculated with the all-treated analysis set as denominator.

^b^Reported in ≥5% of patients.

## Discussion

Results of this first, prospective study of real-life SOC treatment in triple-class exposed patients with RRMM demonstrate poor outcomes with currently available treatments and confirm rapid disease progression after application of salvage therapy. ORR (29.8%) was low, and median PFS (4.6 months) and median OS (12.4 months) were short for these patients. None of the patients evaluated reached sCR and only 1 patient achieved CR, indicating responses were not deep. Responses were not durable, particularly for patients that did not achieve VGPR, who had a median DOR of 4.5 months (95% CI: 3.5–7.3). At the time of enrollment in the study, the majority of patients (74%) were refractory to three classes of antimyeloma drugs, which appeared to be an important prognostic factor of worse outcomes with current SOC treatments. This was indicated by median PFS with SOC treatment of 3.9 months (95% CI: 3.4–4.6) in patients who were triple-class refractory at baseline and 8.2 months (95% CI: 5.7–12.0) in patients who were not triple-class refractory. These data are consistent with several retrospective studies in heavily pretreated patients with RRMM, including the MAMMOTH study, that have generally shown low OS rates and rapid disease progression [3, 7, 8]. Poor outcomes in triple-class exposed patients demonstrate an unmet need for improved treatments in this heavily pretreated group.

As evidenced by the 92 combinations of SOC treatments received by patients, there is not a clearly defined SOC for triple-class exposed patients in real-world practice. This lack of SOC therapy not only leaves patients with few options for well-established treatment, but also complicates the design of clinical trials to compare SOC with new treatments. Data from this study may serve as a benchmark for future comparisons with emerging therapies, as has been the case for the MAMMOTH study in patients who were refractory to anti-CD38 mAbs, which has been used as an indirect comparator against clinical trials lacking a direct comparator arm [9, 10].

One limitation of the observational nature of the LocoMMotion study is that the incidence of TEAEs was likely underestimated. While TEAEs within routine clinical practice were common, occurring in 83.5% of patients, with about half of patients (52.8%) experiencing grade 3/4 TEAEs, it is likely that this is an underestimation that may be attributed to the tendency for physicians to more frequently report adverse events that are clinically relevant or require prescription of additional medications. However, the prospective design of the study enabled collection of all available hematology laboratory results, allowing for calculation of toxicity grades based on the National Cancer Institute Common Terminology Criteria for adverse events, providing a more realistic representation of the toxicity observed with SOC treatments.

In summary, the findings of the LocoMMotion study clearly demonstrate that there is no well-established real-world SOC treatment for triple-class exposed patients with RRMM. The SOC treatments currently being utilized result in poor outcomes and often fail to prevent disease progression. Although the SOC for myeloma will continue to evolve, especially as accessibility and use of BCMA-targeting therapies increases, this study highlights the urgent need for new treatment approaches with novel therapies to improve outcomes in this group of patients.

## Supplementary information

Supplementary Appendix
