# Supplementary material for: LocoMMotion: a prospective, non-interventional, multinational study of real-life current standards of care in patients with relapsed and/or refractory multiple myeloma
Source: Leukemia. 2022 Mar 24;36(5):1371–6. doi: 10.1038/s41375-022-01531-2 (PMC9061296; doi:10.1038/s41375-022-01531-2)
Supplement: Supplementary file 1 — Supplementary Appendix [file 41375_2022_1531_MOESM1_ESM.pdf]

SUPPLEMENTARY APPENDIX

Mateos M-V, et al.*

LocoMMotion: a prospective, non-interventional, multinational study of real-life current standards of care in patients with relapsed and/or refractory multiple myeloma

SUPPLEMENTARY RESULTS

Table S1. SOC treatment regimens in patients (excluding subsequent therapy).

SOC treatment	n (%)
Number of regimens	92
Doublet drug combinations	105 (42.3)
Combinations of ≥3 drugs	160 (64.5)
Regimens (given to ≥4 patients)	
Carfilzomib–dexamethasone	34 (13.7)
Pomalidomide–cyclophosphamide–dexamethasone	33 (13.3)
Pomalidomide–dexamethasone	28 (11.3)
Ixazomib–lenalidomide–dexamethasone	14 (5.6)
Panobinostat–bortezomib–dexamethasone	11 (4.4)
Bendamustine–bortezomib–dexamethasone	7 (2.8)
Carfilzomib–cyclophosphamide–dexamethasone	7 (2.8)
Elotuzumab–pomalidomide–dexamethasone	6 (2.4)
Lenalidomide–dexamethasone	6 (2.4)
Doxorubicin–bortezomib–dexamethasone	5 (2.0)
Carfilzomib–lenalidomide–dexamethasone	5 (2.0)
Carfilzomib–pomalidomide–dexamethasone	5 (2.0)
Melphalan	5 (2.0)
Belantamab mafodotin	4 (1.6)
Bendamustine–prednisone	4 (1.6)
Cyclophosphamide–dexamethasone	4 (1.6)

SOC standard of care.

Patients could receive >1 regimen during SOC treatment. Percentages are calculated with the number of patients in the all-treated analysis set as the denominator.

* *Corresponding author*: Maria-Victoria Mateos, Paseo de la Transición s/n, Hospital Clinico Universitario de Salamanca, Department of Hematology, Salamanca 37007, Spain; mvmateos@usal.es.

Table S2. Summary of subsequent antimyeloma therapy.

	N=248
Any subsequent antimyeloma therapy, n (%)	123 (49.6)
1 therapy	78 (31.5)
2 therapies	18 (7.3)
3 therapies	18 (7.3)
>3 therapies	9 (3.6)
Months on subsequent antimyeloma therapy	
Median (range)	2.8 (0.03–12.29)
Therapy, n (%)	
Glucocorticoids	93 (37.5)
Alkylating agents	67 (27.0)
Proteasome inhibitor	54 (21.8)
Immunomodulatory drug	53 (21.4)
Antibody–drug conjugates	37 (14.9)
Anti-CD38 antibodies	21 (8.5)
Anthracyclines	16 (6.5)
Selective inhibitor of nuclear export	14 (5.6)
Topoisomerase inhibitor	10 (4.0)
Anti-SLAMF7 monoclonal antibody	9 (3.6)
Other antineoplastic agent	8 (3.2)
Bispecific antibodies	6 (2.4)
BCL-2 inhibitor	5 (2.0)
Mitotic inhibitor	5 (2.0)
Antimetabolite	3 (1.2)
Histone deacetylase (HDAC) inhibitor	3 (1.2)
Investigational antineoplastic drug	1 (0.4)
Selective immunosuppressant	1 (0.4)
BCMA-targeted CAR-T	0 (0.0)
Number of regimens	99
Regimen[a] (given to ≥4 patients)	
Belantamab mafodotin	31 (12.5)
Pomalidomide–cyclophosphamide–dexamethasone	8 (3.2)
Thalidomide–cyclophosphamide–dexamethasone	7 (2.8)
Carfilzomib–dexamethasone	6 (2.4)
Monoclonal antibodies[b]	6 (2.4)
Bendamustine	5 (2.0)
Isatuximab–pomalidomide–dexamethasone	5 (2.0)
Belantamab mafodotin–dexamethasone	4 (1.6)
Selinexor–bortezomib–dexamethasone	4 (1.6)

Carfilzomib–cyclophosphamide–dexamethasone	4 (1.6)
Carfilzomib–daratumumab–dexamethasone	4 (1.6)
Elotuzumab–pomalidomide–dexamethasone	4 (1.6)
Ixazomib–lenalidomide–dexamethasone	4 (1.6)
Selinexor–dexamethasone	4 (1.6)

BCMA B-cell maturation antigen.

[a]Patients could receive >1 regimen.

[b]Monoclonal antibodies received by 6 patients in subsequent lines were investigational bispecific antibodies: teclistamab, talquetamab, and AMG701.

Table S3. Cytopenic TEAEs based on adverse event reporting and laboratory values.

Cytopenic TEAE	Total reported (N=248)		Based on laboratory data during SOC therapy (N=244)
	Any grade n (%)[a]	Grade 3/4 n (%)[a]	Grade 3/4 n (%)[b]
Total patients with cytopenic TEAE	106 (42.7)	85 (34.3)	158 (64.8)
Anemia	64 (25.8)	27 (10.9)	46 (18.9)
Thrombocytopenia	57 (23.0)	44 (17.7)	76 (31.1)
Neutropenia	39 (15.7)	33 (13.3)	62 (25.4)
Leukopenia	18 (7.3)	12 (4.8)	58 (23.8)
Lymphopenia	16 (6.5)	14 (5.6)	97 (39.8)

SOC standard of care, TEAE treatment-emergent adverse event.

[a]Percentages are calculated with the all-treated analysis set as denominator.

[b]Percentages are calculated with the number of all treated patients with post-baseline laboratory data as the denominator.
